# Determination of virulence and antibiotic resistance pattern of biofilm producing *Listeria* species isolated from retail raw milk

**DOI:** 10.1186/s12866-016-0880-7

**Published:** 2016-11-08

**Authors:** Kamelia M. Osman, Ahmed Samir, Usama H. Abo-Shama, Essam H. Mohamed, Ahmed Orabi, Tara Zolnikov

**Affiliations:** 1 Department of Microbiology, Faculty of Veterinary Medicine, Cairo University, PO Box 12211, Cairo, Egypt; 2 Department of Microbiology, Faculty of Veterinary Medicine, Sohag University, Sohag, Egypt; 3 Department of Bacteriology, Mycology and Immunology, Faculty of Veterinary Medicine, Zagazig University, Zagazig, 44519 Egypt; 4 North Dakota State University, Developmental Science, Fargo, ND 58102 USA

**Keywords:** *Listeria* species, Buffalo, Cow, Virulence genes, Biofilm formation, Antibiotic resistance

## Abstract

**Background:**

One of the foodborne pathogens is *Listeria monocytogenes*, which causes serious invasive illness in elderly and immunocompromised patients, pregnant women, newborns and infants ranking second after salmonellosis because of its high case fatality rate. Listerial cow mastitis marked by abnormal milk, increased cell counts and reduced production has not been reported. Therefore, apparently healthy cows can be reservoirs of *L. monocytogenes*. A number of 203 udder milk samples from apparently healthy animals (buffalo, *n* = 100; cow, *n* = 103) were collected and tested for *Listeria*. Isolated colonies on the PALCAM agar were *Listeria* species confirmed according to their biochemical and the Christie–Atkins–Munch-Petersen (CAMP) reactions. The *Listeria* species pathogenicity of was tested by phosphatidylinositol-specific phospholipase C, DL-alanine-β-naphthylamide HCl, Dalanine-*p*-nitroanilide tests, chick embryo, mice inoculation tests, Vero cell cytotoxicity and biofilm formation. The virulence-associated genes, *hly*A, *plc*B, *act*A and *iap* associated with *Listeria* were molecularly assayed.

**Results:**

The 17 isolated *Listeria* spp. represented a prevalence rate of 8.4 %. Of these 3 (1.4 %), 2 (1 %), 5 (2.5 %), 4 (2 %) and 3 (1.5 %) were confirmed as *L. monocytogenes, L. innocua, L. welshimeri*, *L. seelegeri*, respectively. While the *L. monocytogenes* isolate displayed all the four virulence-associated genes, *L. seelegeri* carried the *hly*A gene only. The *L. monocytogenes* had a strong in vitro affinity to form a biofilm, in particular serotype 4 which is associated with human infections. *L. monocytogenes* showed resistance for 9/27 antibiotics.

**Conclusions:**

The biofilm forming capability of the *Listeria* spps. makes them particularly successful in colonizing surfaces within the host thus being responsible for persistence infections and due to their antimicrobial resistant phenotype that this structure confers. In addition, strains belonging to serotypes associated with human infections and characterized by pathogenic potential (serotype 4) are capable to persist within the processing plants forming biofilm and thus being a medical hazard.

## Background

The Egyptians kept cattle [1] and milking pictures are as old as 6000 y in Egypt and Iraq [2]. In Egypt, Livestock numbers in 2009 recorded 4.00 million buffaloes and 5.00 million cattle [3]. Cattle are one of the oldest domesticated animals. Their uses lies in transportation, as a food, and fiber for clothing and significant milk producers whose milk and milk products are an important component of the human diet globally.

*Listeria* spp*.* are microaerophilic, belonging to the Firmicutes phylum. Members of the *Listeria* genus are— *L. monocytogenes*, *L. ivanovii*, *L. innocua*, *L. seeligeri*, *L. welshimeri*, *L. grayi*, *L. marthii* and *L. rocourtiae* [4]. While *L. monocytogenes* is pathogenic for humans and animals, *L. ivanovii* is pathogenic to ruminants, and the other species are nonpathogenic [4].

Listeriosis affects domestic and wild animals, most often sheep and cattle, rarely goats, horses and poultry. Buffaloes and cows can excrete *Listeria* after miscarriage or during udder infections followed by mastitis through milk [5]. Cows with listeric mastitis may produce normal-appearing milk containing large numbers of bacteria [6]. This can be manifested in some cases for several years. Cow milk is mentioned as carrier of the fatal listeriosis [7]. Although listerial intramammary infections are rare, identification of infected animals is necessary for the dairy industry since contaminated milk and milk products have been involved in several outbreaks of listeriosis [7–10]. At the same time, serious concerns about the resistance of foodborne pathogens to antibiotics have been growing for a number of years at both national and international levels. Resistance of bacterial pathogens to antibiotics has increased worldwide, leading to treatment failures in human and animal infectious diseases [11].

In Egypt, listerial mastitis in buffalo and cow has not been previously perused. Therefore, we scrutinized carefully in the current study, the prevalence, isolation and identification of the different *Listeria* species as based on selective plating media, biochemical characterization, serological identification, their in vivo virulence potential, antibiotic resistance (ABR) profile and the connection between ABR, biofilm formation and virulence-associated genes (*hly*A, *plc*B, *act*A and *iap*).

## Methods

The study was performed at the Department of Microbiology, Faculty of Veterinary Medicine, Cairo University, Egypt. This work was performed in accordance with the recommendations in the updated Guide for the Care and Use of Laboratory Animals published by the National Institutes of Health (NRC, 2010). All procedures were approved by the Cairo University Ethical Committee in compliance with the United Kingdom (UK) Animals (scientific procedures) Act of 1986, all required approvals were obtained prior to the experiments.

### Sampling

A total of 203 milk samples were collected from buffalo (*n* = 100) and cow (*n* = 103) from private dairy farms around Cairo. The milk and udder were never visibly abnormal throughout the course of the investigation. The animals had not been treated with an antibiotic for at least 30 days prior to collection. A composite milk sample (all four quarters in one collection vial to represent one udder) was taken after gentle stirring [12] under aseptic conditions for bacteriological examinations. A subsample of 15 ml of milk, taken from the composite milk sample, was collected in sterile universal bottles. The milk samples were quickly transported to the laboratory under chilled conditions and stored at 4 °C till bacteriologically analyzed [13–15].

### Isolation and identification of *Listeria* species

For the isolation and identification of *Listeria* species in the milk samples, the techniques previously adopted by Osman et al. [14, 15] were implemented.

### Primary selective enrichment

The primary selective enrichment step involved a selective liquid median Half Fraser Enrichment Broth Antibiotic supplement CCFA (Oxoid; CM0895B and SR0166E). Twenty-five milliliter of each milk sample was added to 500 ml of half Fraser broth, mixed and incubated at 30 °C for 48 h.

### Secondary selective enrichment

From the pre-enrichment culture (half Fraser broth), 0.1 ml was transferred into 10 ml of Fraser broth (Oxoid; CM0895B and SR0156E) and incubated at 35 °C for 48 h.

### Culture and strain characterization

From the culture obtained in Fraser broth, a loopful of the culture was streaked onto PALCAM agar plates (Oxoid; CM0877B and SR0156E) and incubated at 37 °C for 24 to 48 h. The plates were examined for the presence of characteristic colonies presumed to be *Listeria.* Identification of *Listeria* species on PALCAM agar plates was based on aesculin hydrolysis and mannitol fermentation. All *Listeria* species hydrolyse aesculin as evidenced by a blackening of the medium. Mannitol fermentation was demonstrated by a colour change in the colony and/or surrounding medium from red or gray to yellow due to the production of acidic end products. The selectivity of the PALCAM medium is achieved through the presence of lithium chloride, polymixin B sulphate and acriflavine hydrochloride present in the medium base and ceftazidime provided by PALCAM antimicrobial supplement. These agents effectively suppress growth of most commonly occurring non-*Listeria* species of bacteria present in food samples.

### Confirmation of the isolates

All strains were tested for purity, in addition to morphological and biochemical characteristics. Colonies suspected to be *Listeria* were transferred onto pre-dried plates of trytic soya yeast extract agar (TSYEA) (Difco, Bacton, USA) and incubated at 30 °C for 18 to 24 h. Those putative *Listeria* colonies were characterized using Gram’s staining, tumbling motility at 20–25 °C, catalase test, Methyl Red – Voges Proskauer (MR–VP) reactions, characteristics of haemolysis on 5 % sheep blood agar (SBA), carbohydrate utilization and CAMP test. The CAMP test was undertaken using *S. aureus* (ATCC 7494) and *Rhodococcus equi* (ATCC 6939) and *E. coli* (ATCC 25922). They were streaked in single lines across a sheep blood agar plate so that the two cultures were parallel and diametrically opposite. Test strains were then streaked at right angles and 1 to 2 mm apart to *S. aureus* and *R. equi*. Simultaneously, standard strain of *L. monocytogenes* (ATCC 7494) was streaked onto blood agar plates. The plates were then incubated at 37 °C for 18 to 24 h. The test culture streaks were tested for enhanced β-hemolysis at both ends proximal to the reference cultures. The zone of enhanced β-hemolysis may resemble an arrowhead, circle or rectangle. The presence of this zone indicates a CAMP-positive reaction. Absence of enhanced β-hemolysis indicates a CAMP-negative reaction. *L. monocytogenes* and *L. seeligeri* are CAMP-positive to the *Staphylococcus* reference strain and CAMP-negative to *R. equi*. In contrast, *L. ivanovii* is CAMP-positive to the *R. equi* reference strain and CAMP-negative to the *Staphylococcus* reference strains. For the carbohydrate utilization test, isolated colonies from TSYEA were transferred into test tubes containing xylose, rhamnose and mannitol and incubated at 37^0^C for up to 5 days. Positive reactions were indicated by yellow color (acid formation) and occured mostly within 24 to 48 h. In parallel, strains were identified using the API® *Listeria* system (bioMe’rieux, Marcy l’Etoile, France) and the Oxoid Microbact™ *Listeria* 12L (MB1128A). The Microbact™ *Listeria* 12L system is intended to be used for the identification of *Listeria* spp. isolated from the milk samples.

### Phosphatidylinositol-specific phospholipase C (PI-PLC) assay

All the biochemically characterized *Listeria* isolates were assayed for PI-PLC activity [16]. In brief, the *Listeria* isolates were grown overnight on sheep blood agar (SBA) plates at 37 °C. The growth of each *Listeria* isolate harvested from the SBA plate was spot inoculated separately on tryptone soya yeast extract agar plates supplemented with 2.5 mM CaCl_2_ and 40 mM MgSO_4_ in a manner to get a clear visible bacterial growth of approximately 2 mm diameter following an incubation at 37 °C for 24 to 48 h. An overlay suspension was prepared and overlayed at a rate of 4 ml per petridish (9 cm Ø) onto the previously seeded colonies of *Listeria* isolates on TSYE. The plates were then incubated at 37 °C and observed daily for turbid halos around colonies up to 5 days.

### DL-alanine-b-naphthylamide and D-alanine-p-nitroanilide hydrolysis tests

The DL-alanine-β-naphthylamide HCl (DLABN) and Dalanine-*p*-nitroanilide (DAPN) tests were performed as described previously [17] in order to differentiate *L. monocytogenes* from other species of *Listeria*. Cultures were grown on TSA or blood agar for 18 to 24 h at 35 °C, and the majority of the growth was harvested with a sterile cotton swab into 1.0 ml of sterile saline, and of this, 25-ml aliquots were added to 96-well microtiter plates. A 20 mM DL-alanine-β-naphthylamide HCl solution was prepared in sterile 0.05 M Tris–HCl buffer (pH 7.0). A 20 mM Dalanine-*p*-nitroanilide (DAPN) solution was similarly prepared. Fast violet B solution (FVB) was prepared by dissolving a 12.0-mg capsule in 5 ml of sterile distilled water. Just prior to the test, the DLABN or the DAPN stock was diluted in 0.05 M Tris–HCl (pH 7.0) to give a 15 mM solution, and 50 ml was added to 25 ml of the bacterial suspensions in the wells of the sterile 96-well microtiter plate. Negative controls contained 25 ml of saline in place of the bacterial suspension. The plate was covered with stretch plastic and incubated in the dark at 37 °C for 4 h for DLABN or overnight for DAPN. The cover was removed, and 50 ml of FVB solution was added to the colorless DLABN tests. Free β-naphthylamine was detected by observing the deep yellow-orange complex, which developed within 5 min. Hydrolysis of the DAPN was seen by the liberation of the intense yellow *p*-nitrophenol.

The presumptive colonies of *Listeria* (at least three per plate) were further confirmed by various methods as described below.

### Serotype profile

Serotyping was carried out on *L. monocytogenes* strains using commercial specific antisera (Behringwerke AG) against the serovars 1 and 4, following the manufacturer’s instructions.

### Antibiogram profile

The 17 purified isolates were tested by the standard disc diffusion method [18] and were subjected to a susceptibility panel of 28 antibiotics (Oxoid) belonging to 11 drug classes. Isolates were cultured in trypticase-soy broth (TSB) supplemented with 0.6 % yeast extract, and transferred to Mueller–Hinton agar (Oxoid). The plates were incubated at 37 °C for 48 h. Antimicrobials were selected for testing based on the licensing for mastitis treatment in cattle, use in human medicine and potential resistant-determinant phenotypes located on genetic mobile elements (TET and ERY) [19, 20]. Resistance was determined by measurement of inhibition of growth around the antimicrobial disk according to the zone diameter interpretative standards of CLSI [18] or according to the antimicrobials manufacturers’ instructions: Penicillins: Ampicillin (25 μg), Penicillin G (10 IU), Amoxicillin/clavulanic acid (10 μg), Cloxacillin (5 μg), Oxacillin (1 μg), Amoxicillin (25 μg); Fluorquinolones: Ofloxacin (10 μg), Enrofloxacin (10 μg), Ciprofloxacin (5 μg), Flumequine (30 μg), Pefloxacin (30 μg); Aminoglycosides: Amikacin (30 μg), Gentamicin (10 μg), Kanamycin (30 μg), Neomycin (10 μg), Streptomycin (10 μg); Cephalosporins: Cefotaxime (30 μg), Cephalothin (30 μg); Lincosamides: Lincomycin (2 μg), Clindamycin (2 μg); Phenicol: Chloramphenicol (30 μg); Tetracycline: Tetracycline (30 μg); Glycopeptide: Vancomycin (30 μg); Rifamycin: Rifampicin (5 μg); Macrolide: Erythromycin (15 μg), Spiramycin (100 μg); Polypeptides: Bacitracin (10 units); Sulfonamide: Trimethoprim-sulfamathoxazole 1:19 (25 μg).

The isolates were further tested for their pathogenicity. The classical tests for *Listeria* pathogenicity are the Anton conjunctivitis test (rabbits), inoculation of mice, and inoculation of embryonated eggs [13].

### Mice inoculation test

The pathogenicity testing of the *Listeria* isolates by mice inoculation test was performed according to the method described by Menudier et al. [21]. Briefly, the test isolates of *Listeria* were grown on brain heart infusion agar (BHI) slants at 37 °C for 24 h. Female BALB/c mice weighing 18–20 g were housed five per cage and allowed to acclimatize for one week. They were inoculated intraperitoneally with 0.1 ml of inoculum having approximately 10^9^ CFU of each *Listeria* isolate/ml. One group of five mice was injected with 0.1 ml sterile saline, and one group of three mice was not injected. Observations were made daily and mortalities recorded until all of the mice inoculated with virulent strain EGD (NCTC7973) had died. One dead mouse per group was necropsied and spleen recovered for listerial isolation in BHI agar and subsequent PCR confirmation. On the 15th day after inoculation, all surviving mice were euthanized, and one mouse per group was necropsied and the livers and spleens were removed and homogenized, and the bacterial load was enumerated by colony counting [22] for *Listeria*.

The CFUs for individual *L. monocytogenes* strains were obtained by plating aliquots of diluted *L. monocytogenes* suspensions (at 10^9^) on BHI agar. After overnight incubation, the resultant colonies were enumerated, and the CFUs in the original inoculations calculated. The 50 % lethal dose (LD_50_) for each strain was then determined on the basis of mouse mortality data and CFU. The relative virulence (%) was calculated by dividing the number of dead mice recorded with the number of mice tested per strain using the mortality data of the control strain *L. monocytogenes* EGD (NCTC7973) as reference points.

### Chick embryo inoculation test

The pathogenicity of *Listeria* isolates was assessed by chick embryo inoculation test as per the method described by Olier et al. [23]. Briefly, the blood vessel-free surface of chorioallantoic membrane of 60 pre-candled 12-day old embryonated chicken eggs were inoculated with 0.1 ml of a 10^−4^ dilution of the McF #1 equivalent test culture in Tryptose phosphate broth (TPB). The inoculated eggs along with the controls (eggs inoculated with 0.1 ml of sterile TPB) were incubated horizontally at 37 °C for 5 days and were examined twice a day by transillumination for embryo death, if any. Any test isolate causing embryo mortality after 24 h of inoculation was considered as pathogenic.

### Anton’s eye test

An experimental keratoconjunctivitis test (Anton’s eye test) was performed in rabbits by inoculating a drop of live bacterial suspension of *Listeria* species onto the eye. Only *L. monocytogenes* causes purulent keratoconjuctivitis within 24–36 h of inoculation [24] and usually heals spontaneously [25].

### Vero cell cytotoxicity assay

The cytotoxicity assay on Vero cells was carried out according to Raja et al. [26] obtained from American Type Culture Collection, Rockville, Madison, USA, was used. Cell Free Culture Supernatants (CFCS) of the 17*Listeria* isolates were prepared. Each bacterial strain was grown in BHI broth for 24 h at 37 °C. After incubation, an inoculum containing 5 × 10^7^ CFU/ml was taken from which 100 μl was added to the wells containing Vero cells. The bacterial cells were allowed to infect the epithelial cells for 30 min to 12 h at 37 °C. A control set up was maintained where the cell lines were not treated with the pathogen. Live cells were taken out and the cytological changes were observed using an inverted microscope. The Vero cell-line was maintained at 37 °C (4–6 % CO_2_ and 85 % humidity) in a Vero medium, which was Dulbecco’s minimal essential medium with Earle’s salts, 1 % sodium pyruvate, 20 % fetal bovine serum, 1.0 % nonessential amino acids, 1.5 g/L sodium bicarbonate, and, when appropriate, penicillin G and streptomycin (each at 100 μg/mL). All invasion assays were performed at 37 °C as previously described (Raja et al., 2010). Briefly, 48 h before the assay, Vero cells were seeded into 24-well tissue culture plates at a density of 5 × 10^4^ cells/well in the Vero medium without antibiotics. For infection, ∼2 × 10^7^ CFU of each *Listeria* species isolated were added to each well (representing a multiplicity of infection of ∼200). All inocula were enumerated on BHI agar plates. Thirty minutes postinfection, the Vero monolayers were washed three times with PBS to remove any unassociated *Listeria* species, and the medium was replaced with a fresh Vero medium. Forty-five minutes after infection, the medium was replaced with the Vero medium plus 150 μg/mL gentamycin to kill any extracellular *Listeria* species. At 90 min postinfection, Vero cells were washed three times with PBS and lysed with ice-cold distilled water. Intracellular *Listeria* species were enumerated by plating the appropriate dilutions of the Vero lysate on BHI agar. At least three independent trials of the invasion assays were performed with duplicate wells tested for each treatment in each replicate.

### Biofilm formation

#### Glass test tubes

Biofilm formation assay was determined according to Giacaman et al. [27] by Christensen’s tube method. Each *Listeria* strain was inoculated with loopful of microorganism from overnight culture plates in brain heart infusion broth (10 ml) and incubated for 24 h at 37 °C. The cells were incubated in the test tubes without shaking. The tubes were decanted and washed with PBS (pH 7.3) and dried. Dried tubes were stained with crystal violet (0.1 %). Excess stain was removed and tubes were washed with deionized water. Tubes were than dried in inverted position and observed for slime layer formation. Biofilm formation was considered positive when a visible film lined the wall and bottom of the tube. Ring formation at the liquid interface was not indicative of biofilm formation. All the strains were tested in triplicate. Tubes were examined and the amount of biofilm formation was scored as 0-absent, 1-weak, 2-moderate or 3-strong.

#### Microtiter plate assay

*Listeria* species isolates were recovered from −80 °C glycerol stocks onto tryptic soy agar and were stored at 4 °C. Isolated colonies were used to inoculate 3 ml of tryptic soy broth enriched with 0.6 % yeast extract (TSBYE) in sterile 15- by 100-mm glass culture tubes and were incubated for 24 h at 37 °C. According to the procedure of Borucki et al. [28], one milliliter of a 1:40 dilution of each overnight culture was prepared in freshly made Modified Welshimer’s broth (MWB) and was vortexed for 5 s. One hundred microliters of this dilution was then used to inoculate eight separate wells of a presterilized polyvinyl chloride (PVC) microtiter plate, and eight wells of MWB media were included as a control. The plates were incubated for 40 h at 30 °C. After 40 h the liquid from each of the wells was removed, and unattached cells were removed by rinsing three times in 150 μl of sterile water. Biofilms were stained by adding 50 μl of a 0.1 % crystal violet solution to each well and incubating for 45 min at room temperature. Unbound dye was removed by rinsing three times in 150 μl of sterile water. The crystal violet was solubilized by adding 200 μl of 95 % ethanol and incubating at 4 °C for 30 min. The contents of each well (100 μl) were then transferred to a sterile polystyrene microtiter plate, and the optical density at 595 nm (OD_595_) of each well was measured in a microplate reader.

### Molecular identification

#### DNA extraction

Freshly grown typical *Listeria*-like colonies (black colonies) were collected from the surfaces of Palcam plates and boiled in 400 μl of 1 × Tris-EDTA buffer (pH 8.0) (approximately 10^8^ cells/ml) boiled for 10 min and centrifuged at 14,000 rpm for 10 min to remove denatured proteins and bacterial membranes; 2 μl of the supernatant was used as template for the PCR reaction.

#### PCR detection for the genus *Listeria*

*Listeria* was distinguished on the basis of the 16S rRNA [29] that can be revealed by PCR (forward primer 5-CAG-CMG-CCG-CGG-TAA-TWC-3 where M denotes A or C, and W denotes A or T and reverse primer 5-CTC-CAT-AAA-GGT-GAC-CCT-3) to amplify the 938-bp fragment. Amplification was performed with a volume of 50 μl containing 10 μl (200 ng) of extracted DNA template from bacterial cultures, 5 μl 10× PCR buffer, 0.375 μl MgCl_2_ (1.5 mM), 1.25 μl dNTPs (250 μM), 0.25 μl (1.25 Unit) Ampli Taq DNA polymerase, 0.25 μl (0.5 μM) from each primer pairs. The volume of the reaction mixture was completed to 50 μl using DDW. The reaction mixture was overlaid with mineral oil, and the tubes were placed in a DNA thermal cycler (Perkin-Elmer Cetus, Norwalk, Conn.). The samples were subjected to an initial denaturation step of 94 °C for 4 min, followed by 25 amplification cycles of 1 min at 94 °C (denaturation), 1 min at 60 °C (primer annealing), and 1 min at 72 °C (primer extension) followed by a final extension step of 72 °C for 5 min. PCR reaction products were separated on 1.5 % agarose gels, stained with ethidium bromide and visualized. *L. monocytogenes* strain (ATCC 7494) and an *E. coli* strain (ATCC 25922) were included as positive and negative controls respectively.

#### PCR detection of virulence genes among *Listeria* species

The 17 *Listeria* isolates were screened by PCR for the presence of the virulence genes using the primers: *hly*A (for.) 5′-GCA GTT GCA AGC GCT TGG AGT GAA-3′, (rev.) 5′- GCA ACG TAT CCT CCA GAG TGA TCG-3′ [30] to amplify the 456-bp fragment; *plc*B (for.) 5′-CTG CTT GAG CGT TCA TGTCTC ATC CCC C-3′, (rev.) 5′-ATG GGT TTC ACT CTCCTT CTA C-3′ [15] to amplify the 1484-bp fragment; *act*A (for.) 5′-CGC CGC GGA AATTAA AAA AAG A-3′, (rev.) 5′- ACG AAG GAA CCG GGCTGC TAG - 3′ [31] to amplify the 839-bp fragment; and *Iap* (for.) 5′-ACA AGC TGC ACC TGT TGC AG-3′, (rev.) 5′-TGACAG CGT GTG TAG TAG CA-3′ [32] to amplify the 131-bp fragment. Amplification was performed with a volume of 50 μl containing 10 μl (30–50 ng) of extracted DNA template from bacterial cultures, 5 μl 10× PCR buffer, 5 μl MgCl_2_ (25 mM), 4 μl dNTPs (25 mM), 1.54 μl (2.5 U/ul) Ampli Taq DNA polymerase, 2 μl (0.5 μM) from each primer pairs. The volume of the reaction mixture was completed to 50 μl using Double Distilled Water (DDW). The reaction mixture was overlaid with mineral oil, and the tubes were placed in a DNA thermal cycler (Perkin-Elmer Cetus, Norwalk, Conn.). The samples were subjected to an initial denaturation step of 95 °C for 2 min, followed by 35 amplification cycles of 15 s at 95 °C (denaturation), 30 s at 60 °C (annealing), and 90 s at 72 °C (primer extension) followed by a final extension step of 72 °C for 10 min. Visualization of the PCR products was carried out as previously indicated.

## Results

### Prevalence of *Listeria*

A total of 203 milk samples collected from buffaloes (100) and cows (103) were screened for *Listeria* species (Table 1). The microbiological analysis revealed 17 isolates resembling *Listeria* spp. Three isolates were identified as *L. monocytogenes*. The remaining 14 listeriae were considered *Listeria* spp. The overall prevalences of *Listeria* spp. and *L. monocytogenes* were 6.9 % (14/203) and 1.4 % (3/203), respectively. The prevalence of *Listeria* species was found to be, *L. innocua* (2/203; 1.0 %), *L. welshimeri* (5/203; 2.5 %), *L. seeligeri* (4/203; 2.0 %) and *L. grayi* (3/203; 1.5 %) of the isolates. *L. monocytogenes* was detected in udder milk samples at prevalences of buffalo 1 % (1/100) and 2 % for cow (2/103).

**Table 1:** Pathogenicity profiles of *Listeria* species isolated from buffalo and cow milk

Source of milk samples	*Listeria* species	Serotype	Pathogenicity profile									
			CAMP (+/-) with S/R	PI-PLC	DLABN	DAPN	Anton’s eye test	Mice lethality	Chick-embryo lethality	Vero cell ingestion assay	Biofilm	
											Microtiter plate assay	Christensen’s tube
(ATCC 7494)	*monocytogenes*	4	+/-	+	-	-	+	+	+	++	Strong (O.D. = 0.158)	Strong
Buffalo	*monocytogenes*	1	+/+	+	-	-	+	+	+	++	Strong (O.D. = 0.148)	Moderate
	*innocua*		-	-	+	+	-	-	-	-	Strong (O.D. = 0.167)	Strong
	*seelegeri*		-	-	+	+	-	-	-	-	Strong (O.D. = 0.144)	Moderate
	*seelegeri*		-	-	+	+	-	-	-	-	Strong (O.D. = 0.157)	Moderate
	*welshemeri*		-	-	+	+	-	-	-	-	Strong (O.D. = 0.145)	Weak
	*welshemeri*		-	-	+	+	-	-	-	-	Strong (O.D. = 0.141)	Weak
	*grayi*		-	-	+	+	-	-	-	-	Weak (O.D. = 0.0995)	Moderate
	*grayi*		-	-	+	+	-	-	-	-	Strong (O.D. = 0.134)	Moderate
Cow	*monocytogenes*	4	+/-	+	-	-	+	+	+	++	Strong (O.D. = 0.123)	Strong
	*innocua*		-	-	+	+	-	-	-	-	Strong (O.D. = 0.125)	Strong
	*seelegeri*		-	-	+	+	-	-	-	-	Weak (O.D. = 0.074)	Moderate
	*seelegeri*		-	-	+	+	-	-	-	-	Very Strong (O.D. = 0.231)	Moderate
	*welshemeri*		-	-	+	+	-	-	-	-	Strong (O.D. = 0.128)	Moderate
	*welshemeri*		-	-	+	+	-	-	-	-	Strong (O.D. = 0.121)	Moderate
	*welshemeri*		-	-	+	+	-	-	-	-	Strong (O.D. = 0.117)	Moderate
	*L. grayi*		-	-	+	+	-	-	-	-	Strong (O.D. = 0.173)	Weak

CAMP Christie, Atkins, Munch-Petersen test, *S/R Staphylococcus aureus*/*Rhodococcus equi*, *PI-PLC* phosphatidylinositol-specific phospholipase C, *DLABN* DL-alanine-b-naphthylamide HCl, *DAPN* D-alanine-p-nitroanilide, *O.D.* Optical Density, O.D. _595_ < 0.1 = Weak; O.D. _595_ ≤ 0.1 = Strong; O.D. _595_ > 1 = Very Strong

The three *L. monocytogenes* isolated from 203 milk samples (1.5 %) were serologically typed as follows: The identified *L. monocytogenes* serotypes were Type 1 and this was isolated from the buffalo milk samples while Type 4 *L. monocytogenes* was isolated from samples obtained from cow milk samples only.

### Pathogenicity testing

The pathogenicity testing of the 17 *Listeria* isolates via the PI-PLC, DLABN and DAPN assays, as well as in vivo tests, namely chick embryo, mice inoculation tests, Anton’s eye test and Vero cytotoxicity assay, indicated that, the three hemolytic *L. monocytogenes* isolates were found to be pathogenic (Table 1). Spleens from mice that died during the challenge contained viable *L. monocytogenes* that was recovered on BHI agar and confirmed by PCR. On the other hand, spleens from mice that survived the *Listeria* spp. challenge by day 15 had no viable *Listeria* detectable on BHI agar. All other *Listeria* spp. isolates were non-pathogenic (Table 1). The three hemolytic isolates of *L. monocytogenes* showed the characteristic enhancement of hemolytic zone with *S. aureus* (Table 1).

### Biofilm formation

#### Biofilm formation by *Listeria* strains in glass test tubes

The results of adherence assay to test glass tube assessed by 0.1 % Crystal Violet stain showed that the three isolated *L. monocytogenes* isolates (buffalo and cow) were able to strongly form biofilm on glass surface while the isolated strain from buffalo was moderately adherent (Tables 1 and 2). The *L. innocua*, *L. welshimeri*, *L. seeligari* and *L. grayi* were variable in their results (Tables 1 and 2).

**Table 2:**
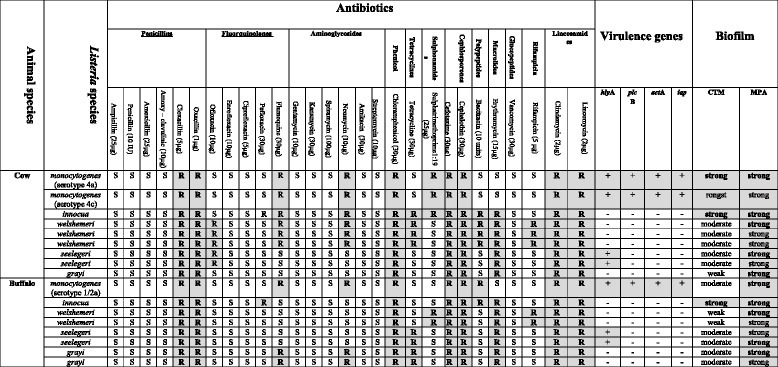
A comparative layout of the diversity in listerial species isolated from buffaloes and cows and their antibiotic resistance phenotype in relation to their affinity to biofilm formation and virulence genes

CTM Christensen’s tube method, *MPA* Microtiter plate assay,  resistant, ***S*** susceptible, Zone diameter, ≤ 18 mm, Resistant; ≥ 25 mm Susceptible

#### Quantitative biofilm formation by *Listeria* strains on polystyrene

The 17 *L. monocytogenes* strains were screened for their adherence to polystyrene 96 well microtiter plates at different degrees. The results showed that *L. monocytogenes* was able to form biofilm on polystyrene (OD_570_ > 1) and was considered as very strong (Tables 1 and 2). The same strong affinity was also observed with *L. innocua*, *L. welshimeri*, *L. seeligari* and *L. grayi* (Tables 1 and 2).

#### Distribution of resistance to individual antimicrobial agents

In this study, the susceptibility of isolates varied between 84.2 and 100 % for most antimicrobials. The major resistance phenotypes was found to be against: cloxacillin, oxacillin, flumequine, neomycin, cefotaxime, cephalothin, lincomycin, clindamycin, chloramphenicol. On the basis of criteria established by the CLSI for *Staphylococcus* species and *Enterococcus* species, the *L. monocytogenes* isolated from the buffalo and cow were resistant to cloxacillin, oxacillin, pefloxacin, flumoquine, cephalosporines, bacitracin, lincomycin and clindamycin. In addition, the *L. monocytogenes* isolated from the buffalo were also resistant to chloramphenicol. On the other hand the *L. monocytogenes* isolated from cow showed resistance for trimethoprim/sulfamathoxazole (Table 2). The other *Listeria* species were diverse in their resistance profile. *L. innocua* isolated from the buffalo and cow were resistant to penicillin, cloxacillins, oxacillins, pefloxacin, flumequine, lincomycins, clindamycin and cephalosporins*.* In addition, the *L. innocua* isolated from bufflo raw milk showed an additional resistance to streptomycins and trimethoprime/sulfamathoxazole. Also, *L. seelegeri* isolates were resistant to cloxacillins, oxacillins, pefloxacin, flumequine, lincomycins and cephalosporins. The *L. welshermeri* isolates were resistant to penicillins, cloxacillins, oxacillins, amoxicillin, ampicillin, amoxicillin, clavulinic acid, pefloxacin, ciprofloxacin, flumequine, lincomycins, and cephalosporins*. L. grayi* isolates were resistant to cloxacillins, oxacillins, pefloxacin, flumequine, lincomycins, clindamycin, cephalosporins and rifampicin.

### Distribution of the virulence genes

For virulotyping analysis, the isolates were screened for the presence of four virulence genes (*hly, plc, act* and *iap)* by conventional PCR which showed that all genes were present in *L. monocytogenes* while absent in other species except *L. seelegeri* which carried the *hly* gene (Table 2).

### Association between antimicrobial resistance, biofilm, serotype and virulence genes

The relationship between antimicrobial resistance, *L monocytogenes* serotypes, affinity for biofilm formation and virulence revealed a strong affinity of the *L. monocytogenes* 1/2a (Lineage II) and 4a and 4c (Lineage III) to form a biofilm on the abiotic surfaces, polystyrene and glass, in addition to their encoding of the virulence genes (*plc*B, *act*A, *hly*A and *iap*) (Table 2). Also, irrespective of the *Listeria* species investigated, 85.7 % (12/14) of the non-*L. monocytogenes* had a strong affinity to form a biofilm on the abiotic polystyrene surface while, only 14.3 % (2/14) of these same species had the same affinity on the glass surface.

### Multiple resistance patterns and distribution

The three *L. monocytogenes* and 14 *Listeria* species displayed multidrug resistance to more than three antibiotics. Their multidrug-resistance patterns are displayed in Table 3. It was noticed that a combination of 14/28 antibiotics were distributed in two forms: cloxacillin/oxacillin/flumequine/pefloxacin/neomycin/cefotaxime/cephalothin/lincomycin/clindamycin/chloramphenicol/tetracycline/erythromycin/bacitracin/trimethoprim-sulfamathoxazole and another combination of cloxacillin/oxacillin/ofloxacin/flumequine/neomycin/cefotaxime/cephalothin/lincomycin/clindamycin/chloramphenicol/tetracycline/rifampicin/erythromycin/bacitracin were the most prevalent and each combination represented 10 classes.

**Table 3:** Antibiotic resistance phenotype combination patterns of different *Listeria* species in cow and buffalo animals

Source of milk	*Listeria* species	Number of *Listeria* species	Patterns of antibiotic combinations	Number of combination patterns	Number of antibiotics	Number of antibiotic classes
Cow						
	*monocytogenes* (serotypes 4a and 4c	2	cloxacillin/oxacillin/flumequine/neomycin/cefotaxime/cephalothin/chloramphenicol/lincomycin/clindamycin/trimethoprim-sulfamathoxazole	5	10/28	7
	*innocua*	1	cloxacillin/oxacillin/flumequine/pefloxacin/neomycin/cefotaxime/cephalothin/lincomycin/clindamycin/ chloramphenicol/tetracycline/erythromycin/bacitracin/trimethoprim-sulfamathoxazole		14/28	10
	*welshemeri*	3	cloxacillin/oxacillin/ofloxacin/flumequine/neomycin/cefotaxime/cephalothin/lincomycin/clindamycin/chloramphenicol/tetracycline/rifampicin/erythromycin/bacitracin		14/28	10
	*seelegeri*	2	cloxacillin/oxacillin/ofloxacin/cefotaxime/cephalothin/lincomycin/clindamycin/chloramphenicol/erythromycin		9/28	6
	*grayi*	1	cloxacillin/oxacillin/cefotaxime/cephalothin/lincomycin/clindamycin/chloramphenicol/erythromycin		8/28	6
Buffalo						
	*monocytogenes* (serotype 1/2a)	1	cloxacillin/oxacillin/flumequine/neomycin/cefotaxime/cephalothin/lincomycin/clindamycin/chloramphenicol	5	9/28	6
	*innocua*	1	cloxacillin/oxacillin/pefloxacin/cefotaxime/cephalothin/lincomycin/clindamycin/chloramphenicol/erythromycin/bacitracin		10/28	7
	*welshemeri*	2	cloxacillin/oxacillin/cefotaxime/cephalothin/lincomycin/clindamycin/chloramphenicol/rifampicin/erythromycin/trimethoprim-sulfamathoxazole		10/28	7
	*seelegeri*	2	cloxacillin/oxacillin/cefotaxime/cephalothin/lincomycin/clindamycin/chloramphenicol/tetracycline/erythromycin		9/28	6
	*grayi*	2	cloxacillin/oxacillin/flumequine/neomycin/cefotaxime/cephalothin/lincomycin/clindamycin/chloramphenicol/tetracycline/erythromycin		11/28	8

The two *L. monocytogenes* isolated from the cow exhibited an antibiotic combination of cloxacillin/oxacillin/flumequine/neomycin/cefotaxime/cephalothin/chloramphenicol/lincomycin/clindamycin/trimethoprim sulfamathoxazole (10/28) representing seven classes. On the other hand, the isolated *L. monocytogenes* isolated from the buffalo was resistant to the cloxacillin/oxacillin/flumequine/neomycin/cefotaxime/cephalothin/lincomycin/clindamycin/chloramphenicol combination (9/28) representing six classes.

## Discussion

Food commodities which can often contain *L. monocytogenes* embrace a large diversification of prepared to-eat or raw foods, equivalent to raw milk or meat and their products, raw mushrooms, soft cheese and seafood [33].

It was essential to distinguish between *Listeria* species and the phylogenetically related *Brochothrix,* which is considered to be of relevance in food spoilage and distinguishable from *Listeria* by being non-motile, non- pathogenic and inability to grow at 35 °C [13]. Our biochemical reactions were consistent with the previous recorded findings of Hitchins and Jinneman, (2013) where *L. monocytogenes* and *L. seeligeri* were hemolytic in sheep blood. The failure of *L. monocytogenes* in the utilization of xylose and ability to utilize rhamnose was also observed by the non-hemolytic species *L. innocua* and *L. grayi* although they were negative in the CAMP test [13]. It should be emphasized that, *L. innocua* sometimes is unable to utilize rhamnose [13] to be interestingly observed with *L. welshimeri* and *L. grayi*. The in vivo pathogenicity assays, (chick embryo, mice inoculation tests, Anton’s eye test and Vero cytotoxicity), confirmed the pathogenicity of the three *L. monocytogenes* isolates and the non-pathogenic nature of the other *Listeria* species (*L. innocua*, *L. welshimeri*, *L. seeligeri* and *L. grayi*).

Consequently, moving to the stage of sero- and geno-typing of *Listeria* isolates becomes relevant. The main virulence determinant, Listeriolysin O (LLO), prevails in *L. monocytogenes* coded by the *hly*A gene. According to the *hly*A gene of *L. monocytogenes* the PCR assay could detect 2 cells in milk [34]. Fortunately, *L. ivanovii* was not isolated in our study although being incriminated to be a predominant animal pathogen affecting ruminants [29, 35].

The essential characteristics of bovine [36] and ovine [37] listerial mastitis due to *L. monocytogenes* is subclinical. Several cases of short-lived excretion of *Listeria* bacteria in the milk and carriers not showing any symptoms are referred to in the literature, whereas cases of prolonged mastitis caused by *Listeria*, marked by abnormal milk, increased cell counts and reduced production are not reported [38, 39]. This incriminates cows to be healthy reservoirs of *L. monocytogenes* [40].

Waste– animal or human — are usually used as fertilizers [41]. Plants and vegetables can become contaminated with *Listeria* from septic manure-based fertilizers (www.linguee.fr/anglais-francais/traduction/fertilizers+manure.html *)*. Healthy farm animals, reservoirs of pathogenic *Listeria*, have the potentiality to contaminate meats, dairy products, soil, water, effluents and produce. Also, a recycling condition of listeria strains could be initiated in the farm when the cows shed *L. monocytogenes* back into the environment after drinking contaminated water [33]. The inter- and intra-farm movement of animals and/or farmers is an important contribution in the listeria dispersion throughout the farm [33]. In addition to the feces, [40, 42, 43], animal drinking water [42, 44, 45], feeds or feed components [40, 45], sawdust bedding, farmyard manure, soil in which fodder plants were grown, cattle feed such as silage hay cubes, beet pulp and wheat lees [46], pest and wildlife could disseminate *L. monocytogenes* in their feces [33, 47, 48] are possible sources and routes of *L. monocytogenes* infection for the animals. The success in the control of the previous sources of animal, farm or/and environmental contamination with *L. monocytogenes* would thereby be in the welfare of the farm animals and farm environment. The faecal excretion of pathogenic *Listeria* is a predisposing element in the ascending infection of the mammary gland, a feature previously recorded to occur with *E. coli* or *Streptococcus uberis* [49]. Despite the inescapable presence of *L. monocytogenes* in the environment, listerial bovine mastitis due to *L. monocytogenes* is a rare incidence and the organized studies on this topic is limited [38, 39, 50–55].

Another potential explanation for the presence of persistent strains of *L. monocytogenes* in the farm or/and milk line during the course of our investigation could be due to the residence of mastitic cows and/or a dwelling biofilm in milking machinery and utensils [33]. As shown in the present investigation, the ability of *L. monocytogenes* to produce biofilms is influenced by serotype [56]. Our results revealed a new interesting and additional observation not previously recorded to *Listeria* species, in the strong affinity of the *L. monocytogenes* 1/2a (Lineage II) and 4a and 4c (Lineage III) to form a biofilm on the abiotic, polystyrene and glass surfaces while Kadam et al. [56] and Doijad et al. [57] found serotypes 1/2b and 1/2a as strong biofilm formers while serotype 4b strains did not exhibit strong biofilm formation [56, 57] which could have a drastic outcome in the dairy industry with a consequent hazardous implication on food safety.

Although pathogenicity of *L. monocytogenes* has been related to listeriolysin [58] yet, *L. monocytogenes* phospholipase (PI-PLC) is also a crucial causal element of pathogenicity [15, 59, 60] and has been found to be a well grounded indicator for differentiation between the pathogenic and nonpathogenic *Listeria* species [15]. The pathogenicity of *L. monocytogenes* induced by the PI-PLC is based on the common regulation of the *hly*A gene and the *plc*B gene by the *prf*A encoded protein [61].

Regulations and practices in the use of antibiotics in the veterinary practice vary widely around the globe and are under the influence of the economic and social circumstances [62]. Clinically, it is of significance to point out that the use of the disc diffusion technique, as implemented in this investigation, is an established universal laboratory assay to determine the susceptibility/resistance of pathogenic bacteria, to prescribe the right remedy [63]. Several factors contribute to this global discrepancy between researchers in their results [64–76]: i) Variations in the formulation of trials, experiments and adopted procedures, ii) The unnecessary use of antibiotics by veterinarians unable to reach a correct diagnosis, iii) No accurate global map for national and international antimicrobial consumption rates, iv) The rationale use of antibiotics and lack of effective antibiotic stewardship programmes, iii) Variations in antibiotic consumption, both between and within countries, v) Non-prescription antibiotic use, veterinarians might not adequately screen for appropriate use, vi) Differences between countries that have and have not implemented comprehensive national strategies in controlling resistance, vii) Poor performance of molecular tests (screening *vs* diagnosis, symptomatic *vs* asymptomatic, active *vs* latent infection), setting (low *vs* high prevalence), complexity of test (done by trained *vs* unskilled staff), and comparator (more or less sensitive than the comparator test), viii) The over the counter purchase of antibiotics used for growth promotion bypassing veterinary superviosion, ix) Unjudicious utilization of antimicrobials in livestock rearing. Interestingly, in Egypt animal farms are quite close to humans, and therefore the hypothesis that ecological resistance close to human settlements is anthropogenic in origin must be taken into consideration [62]. It is also of importance to point out that, recording of resistance by the disc diffusion is measured qualitatively thus, any comparison with studies that use other methods of susceptibility testing should not be taken seriously and therefore un-justifiable [77].

The PCR findings are consistent with previously documented research of *hly*A gene [30], *plc*B gene [15], *iap* gene [32] and *act*A gene [31] with the respective sets of primers giving no cross-reactions with other bacteria. The PCR employed in our study proved to be specific for distinguishing the four virulence-associated determinants, *plc*B, *act*A, *hly*A and *iap* endowed in *Listeria* spp isolated from the buffalo.

## Conclusion

Pregnant women should not help with calving or milking that have recently given birth or touch the after birth or come into contact with newborn calves as these are potential sources of *Listeria*. Farmers with deficient immunity are also advised to take the same precautions. Considering this information together with the low but constant level of contamination of bulk milk by *L. monocytogenes* infected cows, it seems likely that *L. monocytogenes* in cows may be transferred to humans by raw milk or milk that have not been correctly pasteurized. The maintainance of strict hygienic conditions and application of raw milk pasteurization is a must to reduce the risks of human infection with *L. monocytogenes.*
